# Construction and validation of a prognostic model for seroma after breast cancer surgery: a dual-center retrospective cohort study

**DOI:** 10.3389/fonc.2026.1645783

**Published:** 2026-05-13

**Authors:** Zongxin Chen, Qian Zhou, Xinyue Zhang, Chaoming Li, Siyu Li, Guanghua Yang, Xiaohong Xue, Bin Zhao

**Affiliations:** 1 Department of General Surgery, Seventh People’s Hospital of Shanghai University of Traditional Chinese Medicine, Shanghai, China; 2 Department of Breast Surgery, Yueyang Hospital of Integrated Traditional Chinese and Western Medicine, Shanghai University of Traditional Chinese Medicine, Shanghai, China

**Keywords:** breast cancer, clinical prediction model, nomogram, risk factors, seroma

## Abstract

**Objective:**

This study aimed to develop and validate a predictive model for postoperative seroma resolution in patients with breast cancer, thereby providing an evidence-based tool for individualized postoperative management.

**Methods:**

This was a dual-center retrospective cohort study. A total of 373 patients who underwent surgery for breast cancer were included and an independent validation cohort(n=84)according to study center. Candidate predictors were screened using univariable and multivariable Cox proportional hazards regression analyses, and a nomogram was subsequently constructed. Internal validation was performed using bootstrap resampling, and model performance was evaluated in terms of discrimination (C-index and time-dependent area under the receiver operating characteristic curve [AUC]), calibration, and clinical utility (decision curve analysis). Validation was conducted in an independent cohort.

**Results:**

Multivariable Cox regression identified four independent predictors: body mass index (BMI), concomitant diabetes mellitus, modified radical mastectomy, and the number of lymph nodes dissected. A nomogram incorporating these variables was established. Following 1, 000 bootstrap resamples in the training cohort, the model yielded a concordance index (C-index) of 0.76 (95% CI, 0.73–0.80). The model demonstrated good discriminatory ability in both the training and validation cohorts. In the training cohort, the AUC was 0.79 (95% CI, 0.75–0.83) on postoperative day 7 and 0.78 (95% CI, 0.74–0.82) on postoperative day 10. In the independent validation cohort, the corresponding AUCs were 0.90 (95% CI, 0.82–0.97) and 0.86 (95% CI, 0.75–0.96), respectively. Calibration plots showed good agreement between predicted and observed probabilities of complete seroma resolution on postoperative days 7 and 10 in both cohorts. Decision curve analysis further demonstrated that the model provided a meaningful net clinical benefit across a broad range of threshold probabilities at both time points, indicating favorable clinical applicability.

**Conclusions:**

We developed a prognostic model for postoperative seroma resolution that may serve as an effective clinical tool to identify high-risk patients, guide early intervention, and reduce the risk of delayed or refractory seroma resolution.

## Introduction

Breast cancer is the most commonly diagnosed malignancy among women in China (1), and surgery remains a cornerstone of its treatment. Postoperative seroma is the most frequent complication after breast cancer surgery and refers to a localized collection of lymphatic and tissue fluid within the surgical field, resulting in a serous cavity (2) Its occurrence not only delays postoperative recovery and increases healthcare burden, but may also lead to secondary infection, flap necrosis, and even compromised oncologic outcomes due to delayed adjuvant therapy.

At present, a variety of preventive strategies have been proposed, including sclerotherapy, flap fixation sutures, and fibrin sealants, all of which have been extensively evaluated in systematic reviews (3–8). However, the central limitation of current prevention and management strategies lies in the absence of risk-stratified, individualized care. On the one hand, most preventive measures are applied as universal, one-size-fits-all approaches, overlooking the substantial heterogeneity among patients. Although a broad consensus has emerged that prevention is preferable to treatment, the optimal strategy for effectively reducing postoperative seroma remains uncertain. For example, one study suggested delaying postoperative shoulder exercises in overweight patients to reduce seroma formation (9), yet the intervention intensity was not tailored according to individual risk, highlighting the lack of personalized management. Similarly, although high-quality systematic reviews (10) have confirmed the effectiveness of several preventive measures, their recommendations remain confined to general guidance and fail to address a key clinical question: how should the optimal preventive strategy be selected according to patient-specific factors such as BMI, extent of surgery, or comorbidities?

On the other hand, no precise risk prediction tool for individualized postoperative management has yet been established. As early as 2006, Kuroi (11) et al. emphasized the need to evaluate individual risk; however, progress in this area has been slow. Existing studies suggest that some models are limited in clinical utility owing to small sample sizes and the lack of external validation (12). Although both domestic and international studies have investigated the risk factors and prognosis of postoperative seroma after breast cancer surgery, most have been single-center studies with relatively small sample sizes and have focused primarily on risk factor identification rather than the development of a unified and widely accepted prognostic model.

Against this background, the present study retrospectively analyzed clinical data from patients undergoing breast cancer surgery at two hospitals to identify the major factors associated with postoperative seroma resolution. Our aim was to develop and validate a clinically applicable prediction model for seroma resolution, thereby providing an evidence-based basis for early identification of high-risk patients and formulation of individualized postoperative interventions, ultimately reducing the risk of refractory seroma.

## Methods

### Study design

**Figure 1:**
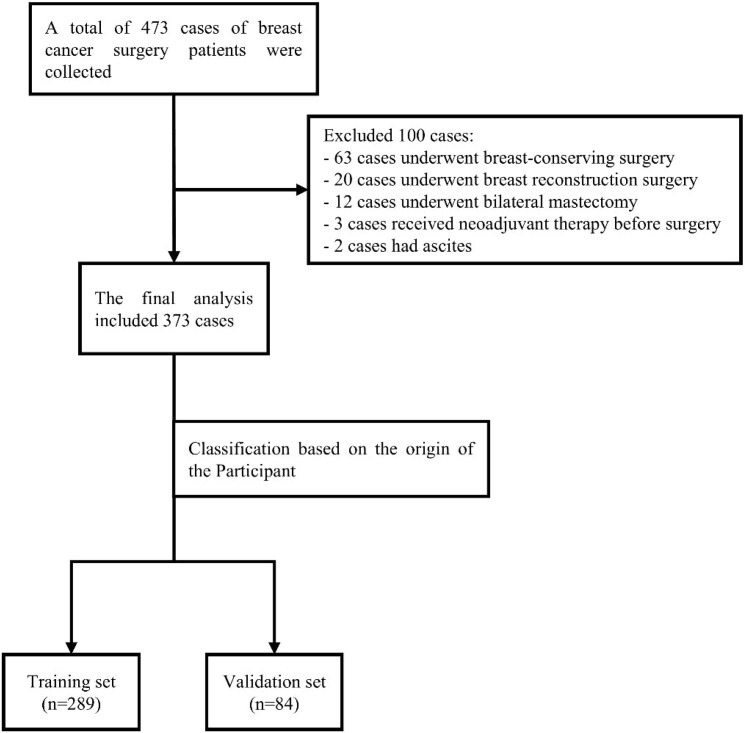
Flowchart of participant screening process.

This was a dual-center retrospective cohort study. Data were obtained from two hospitals. Center 1 was the Department of Breast Disease, Yueyang Hospital of Integrated Traditional Chinese and Western Medicine affiliated with Shanghai University of Traditional Chinese Medicine, where all patients who underwent breast cancer surgery between January 2021 and August 2024 were screened. Center 2 was the Department of Thyroid and Breast Surgery, Shanghai Seventh People’s Hospital, where all patients undergoing breast cancer surgery between January 2022 and August 2024 were screened. A total of 473 patients were initially identified. After application of the eligibility criteria, 373 patients were ultimately included in the statistical analyses. Of these, 289 patients from Yueyang Hospital constituted the training cohort for model development, and 84 patients from Shanghai Seventh People’s Hospital served as the independent validation cohort (**Figure 1**). The study protocol was approved by the Institutional Review Board of Yueyang Hospital (approval No. KYSKSB2020-149) and the Ethics Committee of Shanghai Seventh People’s Hospital (approval No. 2024-7th-HIRB-117). All procedures were conducted in accordance with the Declaration of Helsinki. Because this was a retrospective cohort study based exclusively on routinely collected clinical data, involved no intervention, and all data were anonymized prior to analysis, both ethics committees approved a waiver of informed consent.Both centers followed the National Comprehensive Cancer Network (NCCN) Clinical Practice Guidelines in Oncology for Breast Cancer (2021–2024 editions) and the Chinese Society of Clinical Oncology (CSCO) guidelines for breast cancer diagnosis and treatment (2021–2024 editions) (13).

### Participants

#### Inclusion criteria

(1) Pathologically confirmed primary breast cancer treated with unilateral breast surgery; (2) no neoadjuvant therapy before surgery, including chemotherapy, radiotherapy, or endocrine therapy; and (3) receipt of one of the following definitive procedures: (a) total mastectomy with sentinel lymph node biopsy (TM+SLNB), defined as complete removal of the affected breast tissue with sentinel lymph node biopsy but without systematic axillary lymph node dissection; or (b) modified radical mastectomy (MRM), defined as complete removal of the affected breast, including the nipple–areola complex and overlying skin, together with ipsilateral axillary lymph node dissection, while preserving both the pectoralis major and pectoralis minor muscles.

#### Exclusion criteria

Patients were excluded if they met any of the following criteria: (1) pre-existing ipsilateral upper-extremity lymphedema before surgery; (2) no routine placement of a negative-pressure drainage tube after surgery; (3) acute postoperative bleeding, hematoma, or infection in the flap area unrelated to seroma, precluding accurate assessment of seroma outcome; (4) uncontrolled comorbid conditions, severe organ dysfunction, or severe cardiac, hepatic, or renal insufficiency that could interfere with assessment of seroma prognosis; (5) another primary malignancy, autoimmune disease, or coagulation disorder, a clear contraindication to breast cancer surgery, or any condition that could substantially affect postoperative recovery; or (6) incomplete medical or follow-up records, missing key predictor variables, or inability to definitively determine the seroma outcome.

### Outcome definition

Given the absence of a unified clinical guideline regarding the timing of drain removal after breast cancer surgery, the primary endpoint in this study was defined, according to local clinical practice, as complete seroma resolution. Specifically, nursing records within 14 postoperative days were reviewed. If the drainage volume on any given day—or, after drain removal, the aspirated subflap fluid volume—was first ≤5 mL, that day was defined as the date of complete seroma resolution. The interval from the date of surgery to that date was defined as the time to complete resolution. If the drainage or aspirated volume remained >5 mL on postoperative day 14, the observation was treated as censored at that time point.

### Data collection

The following data were retrieved from the electronic medical record system and postoperative nursing records: (1) demographic and baseline characteristics, including age, body mass index (BMI), presence of diabetes mellitus, and presence of hypertension; (2) surgical and tumor characteristics, including surgical procedure (TM+SLNB or MRM), operative time (hereafter referred to as Op time), maximum tumor diameter, lymph node metastasis (hereafter referred to as LNM), and number of lymph nodes dissected (hereafter referred to as LNs); and (3) outcome-related variables, including daily postoperative drainage volume (mL) recorded up to postoperative day 14, as well as the date and volume of aspiration if needle aspiration was performed after drain removal.

### Handling of missing data

The overall missing-data rate for each variable was less than 5%. Records containing missing values were handled using complete-case analysis.

### Development and validation of the prediction model

#### Model development

All candidate predictors were first subjected to univariable Cox proportional hazards regression analysis. Variables with P<0.10 were entered into the initial multivariable model. Variable selection was then performed using backward stepwise regression based on the likelihood ratio test, with a removal criterion of P>0.10. Variables retained in the final model were required to be both statistically significant (P<0.05) and clinically relevant.

#### Model performance assessment and prespecified time points

To evaluate model performance at clinically relevant decision points, postoperative day 7 (early decision-making) and postoperative day 10 (later management) were prespecified as key time points. Discrimination was assessed using time-dependent receiver operating characteristic (ROC) curves and their corresponding AUCs, calibration was evaluated using calibration plots, and clinical utility was assessed by decision curve analysis. Internal validation was performed using bootstrap resampling with 1, 000 repetitions to correct for optimism, and the optimism-corrected C-index was ultimately reported.

#### Model presentation and assumption testing

The final model was presented as hazard ratios (HRs) with 95% confidence intervals (95% CIs) and visualized as a nomogram. Kaplan–Meier curves were generated for preliminary visualization, and the proportional hazards assumption was rigorously examined using Schoenfeld residual-based tests.

#### Independent validation

To assess model generalizability, the final model was directly applied to the independent validation cohort. Model performance in this cohort was evaluated using the same metrics and procedures as in the training cohort: the C-index and its 95% CI were calculated; time-dependent AUCs and ROC curves were generated for postoperative days 7 and 10; calibration plots were drawn; and decision curve analysis was performed to comprehensively assess discrimination, calibration, and clinical usefulness in a new patient population.

### Statistical analysis

All statistical analyses were performed using R software version 4.4.2. Continuous variables conforming to a normal distribution were presented as mean ± standard deviation and were compared using the t test. Categorical variables are presented as counts (percentages) and were compared using the chi-square test. All hypothesis tests were two-sided, and P<0.05 was considered statistically significant.

## Results

### Baseline characteristics of the study population

A total of 373 patients who underwent breast cancer surgery were included in this study, of whom 289 were assigned to the training cohort. During the 14-day follow-up period, 275 patients (95.16%) in the training cohort achieved complete seroma resolution. Baseline characteristics were well balanced between the training and validation cohorts, with no statistically significant differences observed (**Table 1**).

**Table 1:** Comparison between training set and validation set baselines.

Variables	Total (n = 373)	Training set (n = 289)	Validation set (n = 84)	Statistic	*P*
Age, Mean ± SD	63.08 ± 12.64	63.38 ± 12.23	62.02 ± 13.99	t=0.87	0.386
Op time, Mean ± SD	151.27 ± 54.83	150.44 ± 58.03	154.14 ± 42.17	t=-0.65	0.519
LNs, Mean ± SD	10.33 ± 6.84	10.20 ± 6.90	10.76 ± 6.67	t=-0.66	0.511
BMI, n(%)				χ²=0.50	0.778
18.5-24.9	204 (54.69)	159 (55.02)	45 (53.57)		
<18.5	46 (12.33)	37 (12.80)	9 (10.71)		
>24.9	123 (32.98)	93 (32.18)	30 (35.71)		
Diabetes, n(%)				χ²=0.19	0.661
No	291 (78.02)	224 (77.51)	67 (79.76)		
Yes	82 (21.98)	65 (22.49)	17 (20.24)		
Hypertension, n(%)				χ²=0.12	0.726
No	272 (72.92)	212 (73.36)	60 (71.43)		
Yes	101 (27.08)	77 (26.64)	24 (28.57)		
Surgical approach, n(%)				χ²=2.28	0.131
TM+SLNB	178 (47.72)	144 (49.83)	34 (40.48)		
MRM	195 (52.28)	145 (50.17)	50 (59.52)		
Tumor size, n(%)				χ²=0.22	0.894
T1	166 (44.50)	128 (44.29)	38 (45.24)		
T2	188 (50.40)	147 (50.87)	41 (48.81)		
T3	19 (5.09)	14 (4.84)	5 (5.95)		
Lesion, n(%)				χ²=2.67	0.102
Single	337 (90.35)	265 (91.70)	72 (85.71)		
Multiple	36 (9.65)	24 (8.30)	12 (14.29)		
LNM, n(%)				χ²=0.00	0.995
Negative	253 (67.83)	196 (67.82)	57 (67.86)		
Positive	120 (32.17)	93 (32.18)	27 (32.14)		

t, t-test χ², Chi-square test SD, standard deviation.

BMI, body mass index; HR, hazard ratio; CI, confidence interval.

TM+SLNB, total mastectomy with sentinel lymph node biopsy.

MRM, modified radical mastectomy; LNM, lymph node metastasis.

### Model development and final predictors

Variables with P<0.10 in the univariable Cox regression analyses were entered into the multivariable model. Backward stepwise regression ultimately identified four independent predictors (**Table 2**). Specifically, underweight status, compared with normal weight, was associated with faster resolution and therefore represented a protective factor (HR = 3.37, 95% CI, 2.29–4.96; P<0.001). By contrast, overweight status (HR = 0.66, 95% CI, 0.50–0.88; P = 0.003), concomitant diabetes mellitus (HR = 0.44, 95% CI, 0.32–0.60; P<0.001), modified radical mastectomy (vs TM+SLNB; HR = 0.65, 95% CI, 0.43–0.99; P = 0.043), and a greater number of dissected lymph nodes (per additional node: HR = 0.96, 95% CI, 0.93–0.99; P = 0.027) were all associated with a significantly slower rate of seroma resolution.

**Table 2:** Results of Univariate and multivariate cox regression analysis.

Variables	Univariate Cox regression		Multivariate Cox regression	
	*P*	HR (95%CI)	*P*	HR (95%CI)
BMI				
18.5-24.9		1.00		1.00
<18.5	**<.001**	3.44 (2.37 ~ 5.00)	**<.001**	3.37 (2.29 ~ 4.96)
>24.9	**<.001**	0.50 (0.38 ~ 0.66)	**0.005**	0.66 (0.50 ~ 0.88)
Diabetes				
No		1.00		1.00
Yes	**<.001**	0.37 (0.27 ~ 0.50)	**<.001**	0.44 (0.32 ~ 0.60)
Hypertension				
No		1.00		
Yes	0.294	0.87 (0.66 ~ 1.13)		
Surgical approach				
TM+SLNB		1.00		1.00
MRM	**<.001**	0.40 (0.32 ~ 0.52)	**0.043**	0.65 (0.43 ~ 0.99)
Tumor size				
T1		1.00		
T2	**0.024**	0.76 (0.59 ~ 0.96)		
T3	0.423	0.80 (0.46 ~ 1.39)		
Lesion				
Single		1.00		
Multiple	0.215	1.31 (0.86 ~ 1.99)		
LNM				
Negative		1.00		
Positive	**<.001**	0.53 (0.41 ~ 0.69)		
Age	0.169	0.99 (0.98 ~ 1.00)		
Operative time	**<.001**	0.99 (0.99 ~ 0.99)		
LNs	**<.001**	0.93 (0.91 ~ 0.95)	**0.027**	0.96 (0.93 ~ 0.99)

HR, Hazards Ratio; CI, Confidence Interval.

BMI, body mass index; HR, hazard ratio; CI, confidence interval.

TM+SLNB, total mastectomy with sentinel lymph node biopsy.

LNs, lymph nodes.

MRM, modified radical mastectomy LNM, lymph node metastasis.

Bold values indicate statistical significance (p < 0.05).

### Model performance at key clinical time points

#### Discrimination

**Figure 2:**
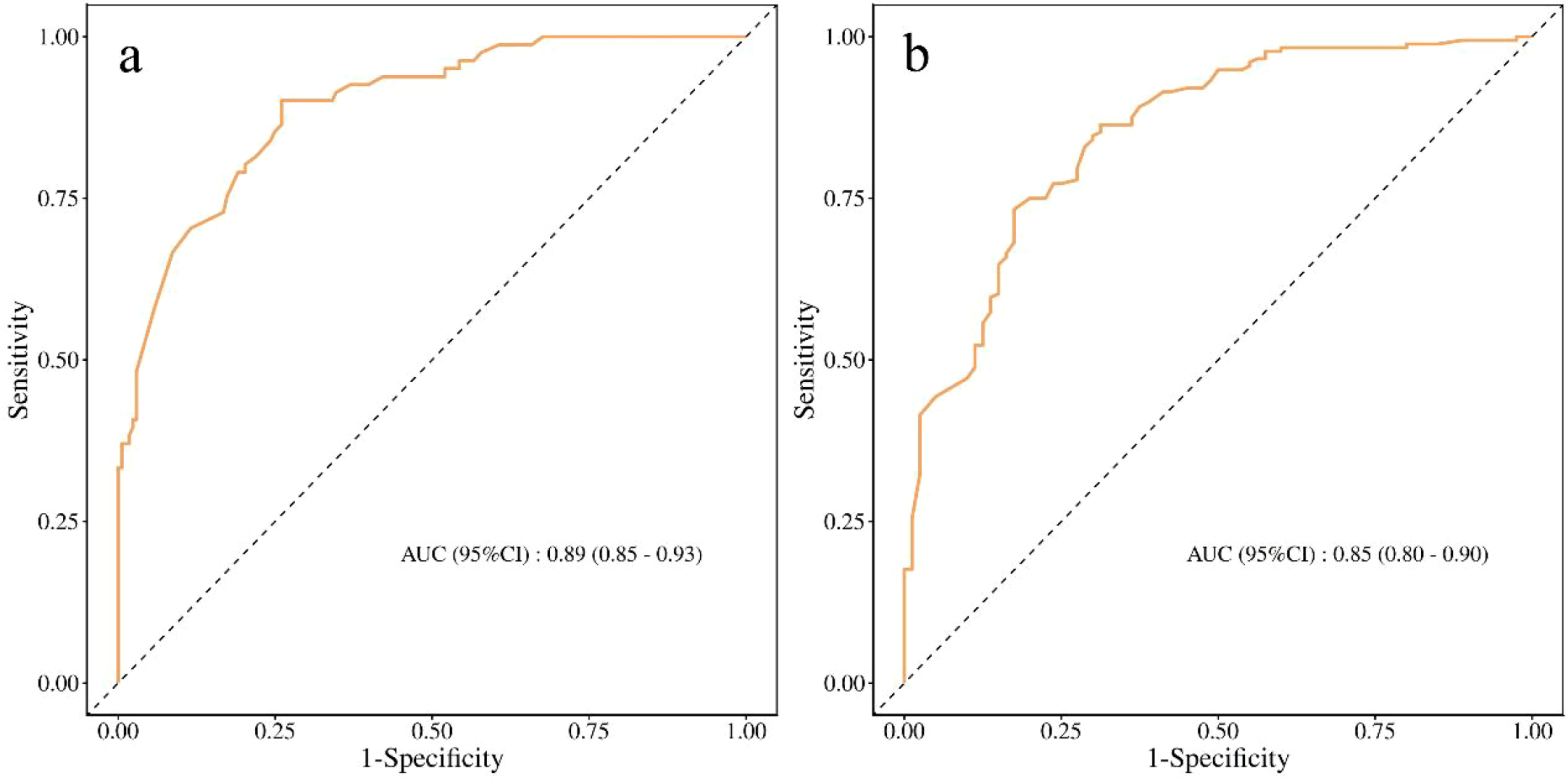
Time-dependent ROC curves of the model in the training cohort at postoperative **(a)** day 7 and **(b)** day 10.

The apparent C-index of the model was 0.77 (95% CI, 0.73–0.80). After bootstrap correction for internal validation, the C-index was 0.76 (95% CI, 0.73–0.80), with an optimism estimate of 0.006. Time-dependent AUC analyses indicated good discrimination on both postoperative day 7 and postoperative day 10 (**Figure 2**). The AUC was 0.79 (95% CI, 0.75–0.83) on day 7 and 0.78 (95% CI, 0.74–0.82) on day 10.

#### Calibration

**Figure 3:**
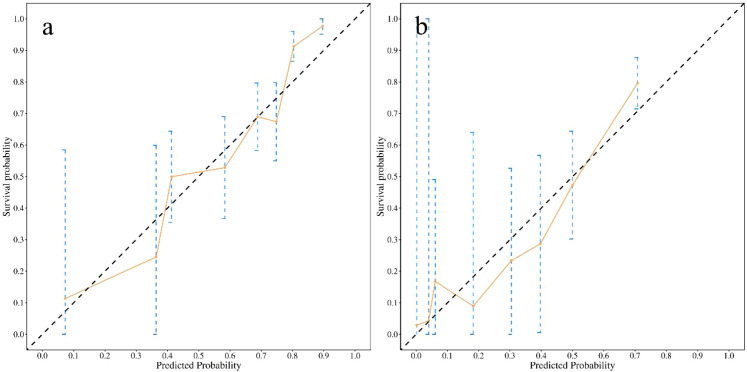
Calibration plots for the training cohort at day 7 **(a)** and day 10 **(b)**. The bias-corrected (via bootstrap) curves closely follow the ideal line, indicating good calibration.

Calibration plots (**Figure 3**) demonstrated good agreement between predicted and observed probabilities of non-resolution at both time points. After 1, 000 bootstrap corrections, the calibration curves remained closely aligned with the ideal diagonal line, indicating high predictive accuracy.

#### Clinical utility

**Figure 4:**
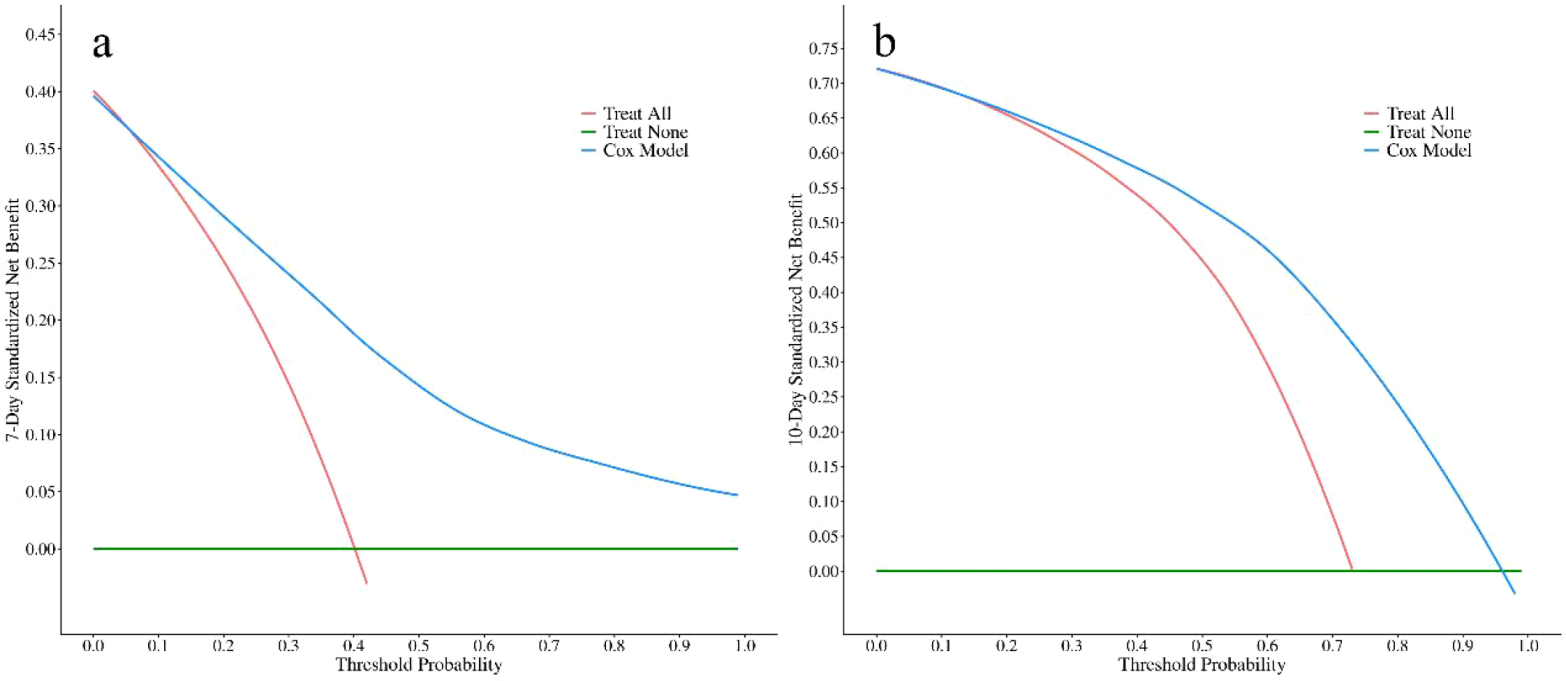
Decision curve analysis at day 7 **(a)** and day 10 **(b)**.​ The net benefit of using the nomogram (blue) is plotted against the “Treat All” (red) and “Treat None” (green) strategies. The model provides a net benefit when the threshold probability is between approximately 15%-100% at day 7 and 20%-95% at day 10.

Decision curve analysis (**Figure 4**) demonstrated excellent clinical utility of the model. As shown, on postoperative day 7, when the threshold probability ranged from 15% to 90%, and on postoperative day 10, when the threshold probability ranged from 20% to 85%, model-guided decision-making consistently yielded a higher net benefit than strategies of intervening in all patients or in none. These findings indicate that the model can provide stable and superior decision support across a wide range of clinical preferences, highlighting its strong translational potential.

### Visualization and interpretation of the prediction model

#### Nomogram

**Figure 5:**
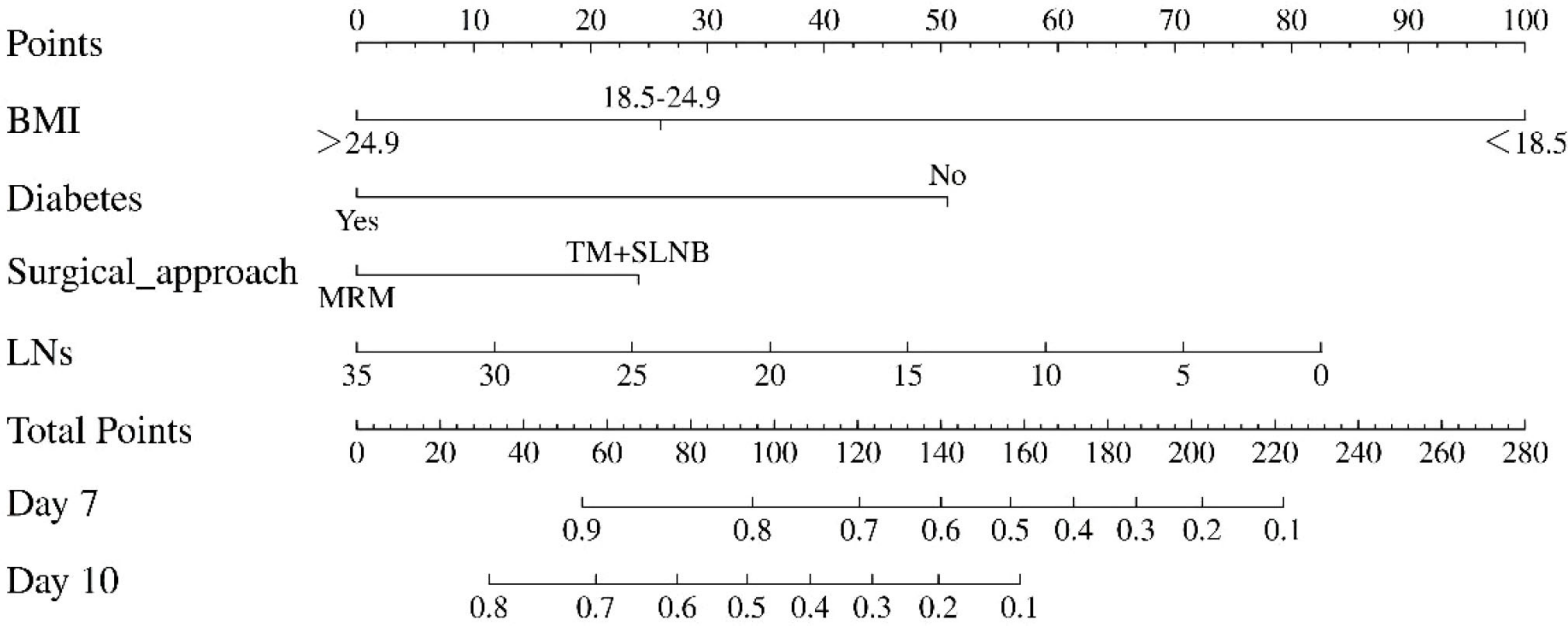
Nomogram for predicting the probability of postoperative seroma non-resolution. This nomogram is designed to individualize the risk assessment of seroma non-resolution at postoperative day 7 and day 10. Instructions for use: 1) For each predictor, locate the patient’s value on the corresponding axis; 2) Draw a line upward to the “Points” axis to determine the score for each variable; 3) Sum all scores to obtain the “Total Points”; 4) Draw a line downward from the “Total Points” axis to the “Predicted Probability” axis to read the estimated probability of seroma non-resolution at postoperative day 7​ and day 10. Note: A higher total points indicates a greater risk of delayed seroma resolution.

A nomogram based on the final model was constructed (**Figure 5**). Clinicians may calculate the total score according to the patient’s four characteristics and directly estimate the predicted probability of non-resolution on postoperative days 7 and 10. For example, for a patient with the following features—overweight (BMI >25 kg/m²), diabetes mellitus, modified radical mastectomy, and 10 dissected lymph nodes—the corresponding probability of non-resolution is approximately 88% on postoperative day 7 and 68% on postoperative day 10.

#### Assessment of the proportional hazards assumption

**Figure 6:**
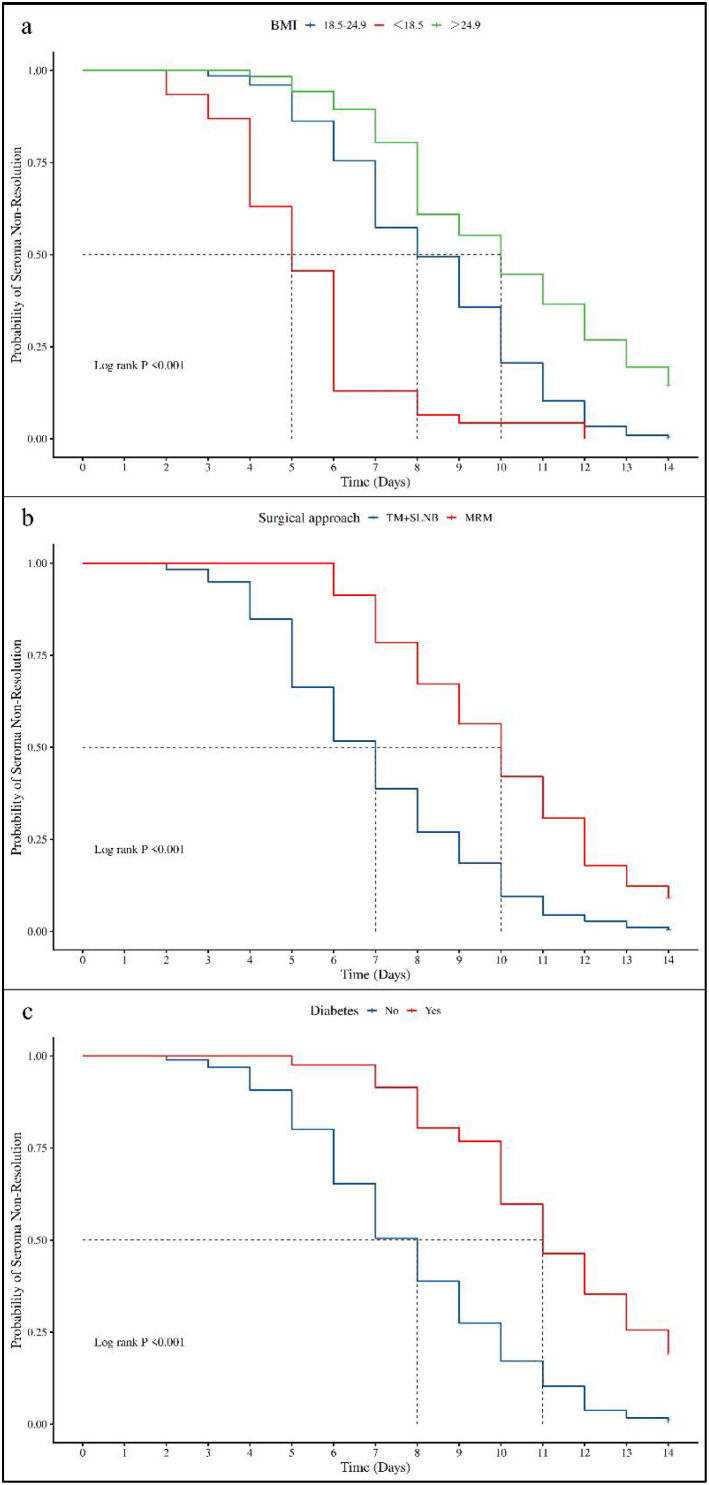
**(a)** Kaplan-Meier curves showing the probability of seroma non-resolution​ over time, stratified by BMI. **(b)** Kaplan-Meier curves showing the probability of seroma non-resolution​ over time, stratified by Surgical approach. **(c)** Kaplan-Meier curves showing the probability of seroma non-resolution​ over time, stratified by Diabetes.

The proportional hazards assumption for the final model was examined using Schoenfeld residual-based tests. All retained variables (BMI, diabetes status, surgical procedure, and number of lymph nodes dissected) yielded P values greater than 0.05, indicating no evidence of violation of the proportional hazards assumption. In addition, Kaplan–Meier curves (**Figure 6**) stratified by BMI, diabetes status, and surgical procedure did not cross and were approximately parallel, providing graphical support for the validity of the proportional hazards assumption.

### Independent validation

**Figure 7:**
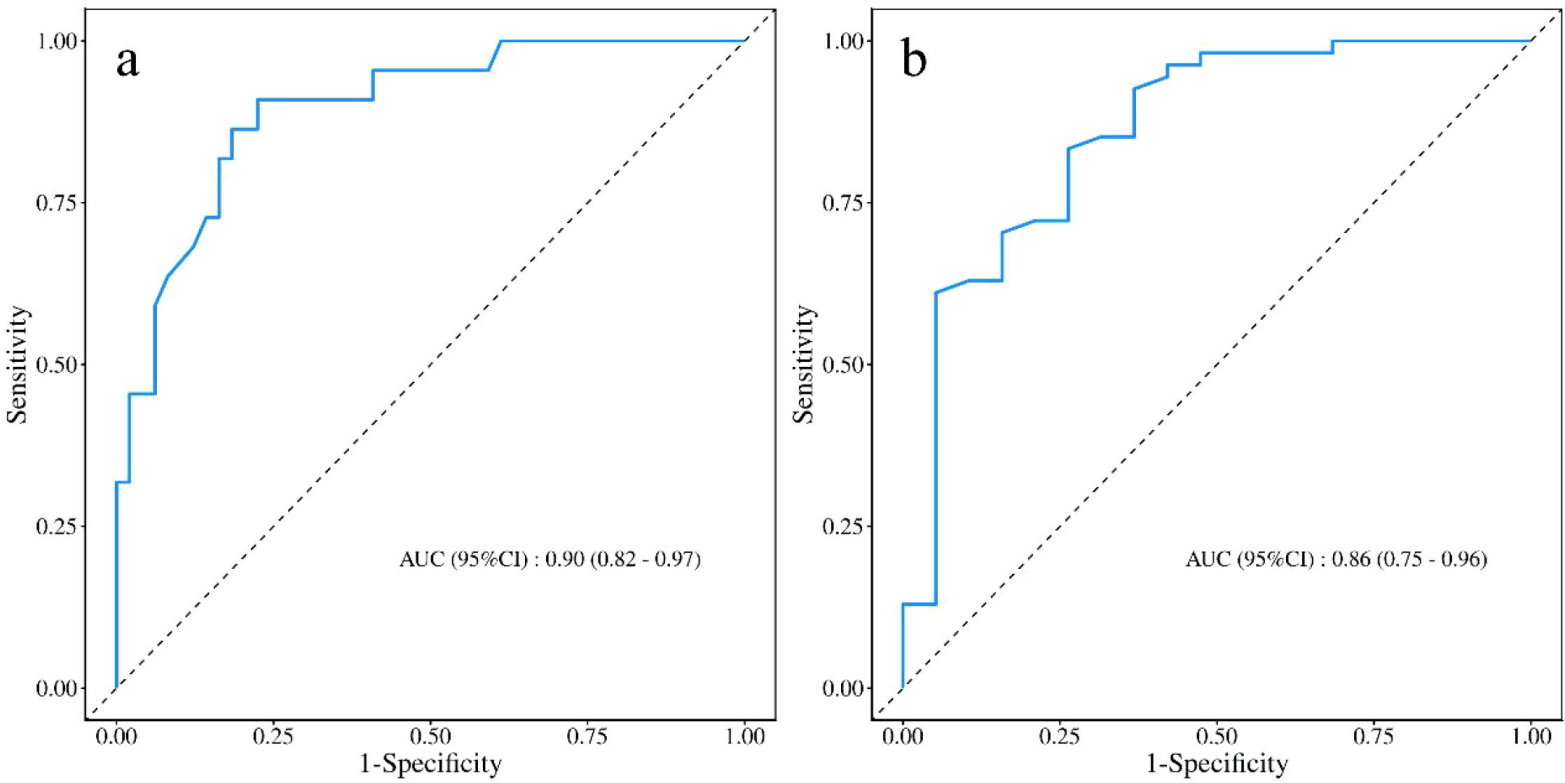
Time-dependent ROC curves of the model in the validation cohort at postoperative **(a)** day 7 and **(b)** day 10.

**Figure 8:**
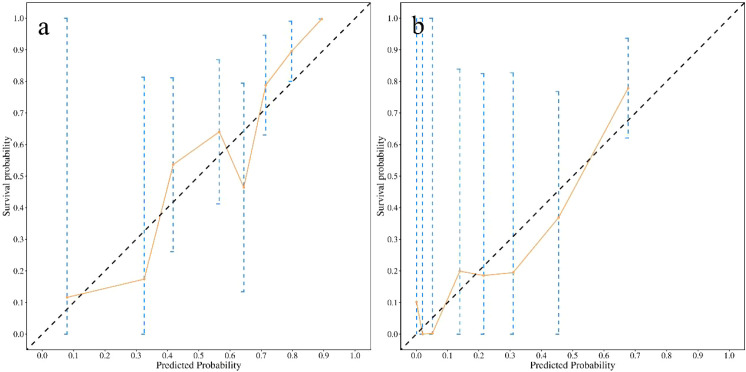
Calibration plots for the validation cohort at day 7 **(a)** and day 10 **(b)**.​ The model demonstrates well-calibrated predictions in the external validation set, as shown by the proximity of the curves to the diagonal.

**Figure 9:**
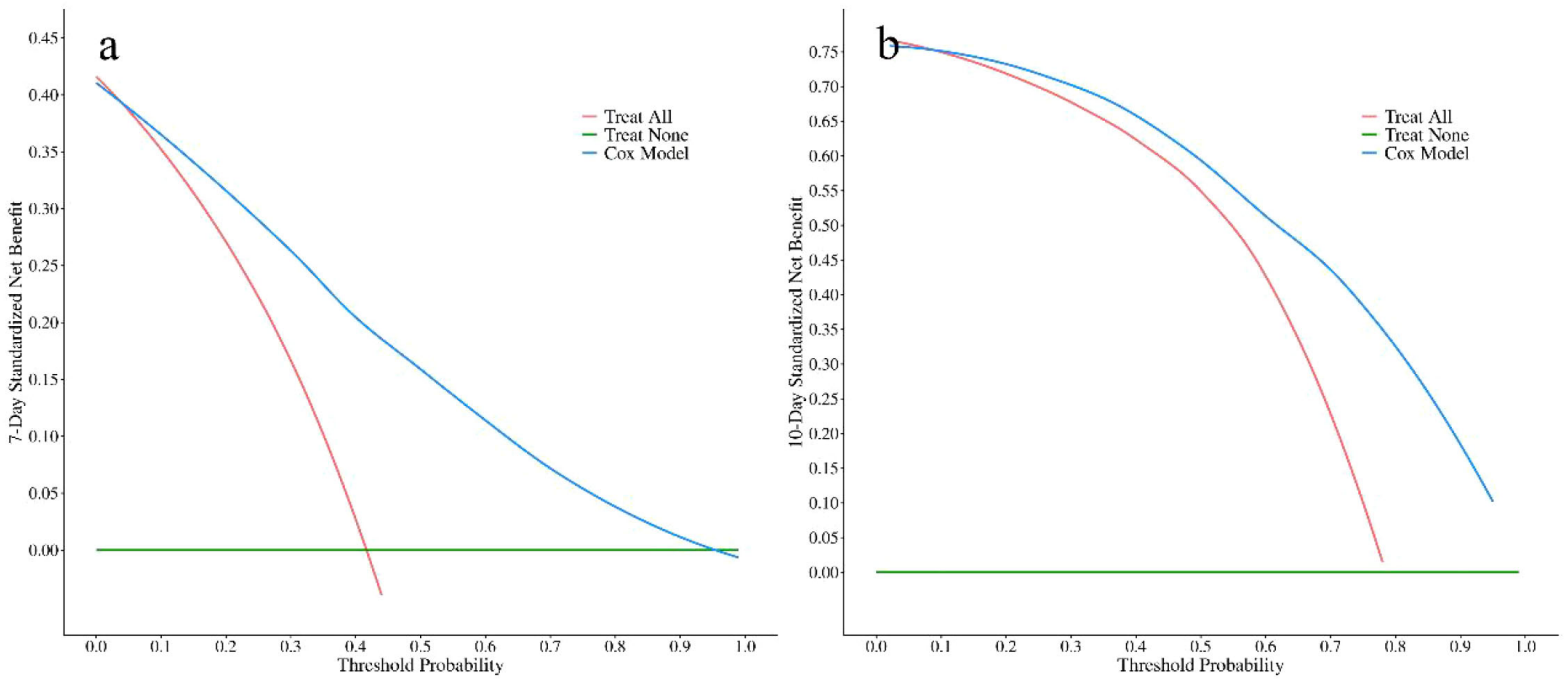
Decision curve analysis at day 7 **(a)** and day 10 **(b)**.​ The net benefit of using the nomogram (blue) is plotted against the “Treat All” (red) and “Treat None” (green) strategies. The model provides a net benefit when the threshold probability is between approximately 5%-95% at day 7 and 10%-95% at day 10.

In the independent validation cohort (n=84), the model maintained good predictive performance. The overall C-index was 0.78 (95% CI, 0.72–0.84), which was comparable to that observed in the training cohort. Time-dependent ROC analysis (**Figure 7**) showed that the AUCs were 0.90 (95% CI, 0.82–0.97) on postoperative day 7 and 0.86 (95% CI, 0.75–0.96) on postoperative day 10, indicating stable discrimination at both key time points. Calibration plots (**Figure 8**) demonstrated good agreement between predicted and observed probabilities at both time points. Decision curve analysis in the independent cohort (**Figure 9**) further confirmed the model’s clinical usefulness. Specifically, on postoperative day 7, when the threshold probability ranged from approximately 5% to 95%, and on postoperative day 10, when the threshold probability ranged from approximately 10% to 95%, the use of the model provided a clear net clinical benefit. These results indicate that the model offers robust and clinically meaningful decision support in an independent patient population across nearly all reasonable clinical preference thresholds.

## Discussion

This study successfully developed and validated a Cox proportional hazards model for predicting the time to postoperative seroma resolution in patients with breast cancer. The model integrates four readily available clinical variables—BMI, diabetes status, surgical procedure, and number of lymph nodes dissected—and demonstrated good discrimination, calibration, and clinical utility. The central finding is that the model not only quantifies each patient’s risk of delayed resolution, but also, more importantly, provides a net clinical benefit when used for risk-stratified management at distinct decision time points (postoperative days 7 and 10), as demonstrated by decision curve analysis.

Our findings indicate that higher BMI, concomitant diabetes mellitus, modified radical mastectomy, and a greater number of dissected lymph nodes together constitute an independent risk profile associated with delayed postoperative seroma resolution. These factors may impair tissue healing, immune function, and lymphatic drainage, thereby slowing seroma resorption. This observation is consistent with previous reports (14–23). More importantly, it suggests that seroma prognosis is jointly determined by baseline patient characteristics and the extent of surgical trauma. Clinically, patients with these high-risk features warrant particular attention, thereby providing a direct rationale for individualized postoperative management based on risk stratification and underscoring the need for an integrated multifactorial prediction model to facilitate precision intervention.

Previous studies have attempted to develop predictive models for seroma. Tansawet et al. (24) used a nine-layer artificial neural network to predict seroma after total mastectomy in a cohort of 120 patients, achieving an AUC of 0.760. However, that model treated seroma as a binary outcome (occurrence vs non-occurrence), did not incorporate the temporal dimension of seroma resolution, and, as acknowledged by the authors, required external validation to confirm its performance. In contrast, the principal innovation of the present study lies in moving beyond the conventional binary framework of ‘resolved’ versus ‘unresolved’ and, to our knowledge, establishing a dynamic prediction model with time to resolution as the endpoint. We systematically evaluated its clinical value at two key decision points—postoperative days 7 and 10—and translated the model into an intuitive nomogram that allows bedside individualized risk assessment, thereby facilitating a seamless bridge from statistical prediction to clinical decision-making.

Decision curve analysis further demonstrated that the model provides a stable net clinical benefit across a remarkably wide range of decision thresholds (day 7: 15%-90%; day 10: 20%–85%). This finding can be directly translated into a pragmatic management pathway. For patients identified as high-risk on postoperative day 7, early proactive intervention may be considered, including ultrasound-guided assessment followed by needle aspiration, adjustment of the drainage tube, or reinforced compression bandaging to actively control seroma progression. For patients who remain high-risk by postoperative day 10, the emphasis should shift toward intensified surveillance and patient education, such as shortening revisit intervals, providing targeted counseling, and closely monitoring fluid accumulation, thereby avoiding overtreatment of patients likely to recover spontaneously while ensuring sustained management of those with delayed recovery. Taken together, as an innovative yet practical decision-support tool, the present model has the potential to shift postoperative seroma management from generalized empirical care toward individualized care based on dynamic risk stratification.

The proportional hazards assumption was rigorously tested in this study, ensuring the reliability of the estimated hazard ratios and strengthening the methodological rigor of the analysis. In addition, by combining bootstrap internal validation with independent external validation, we were able to effectively assess and correct for model overfitting and to report optimism-corrected performance metrics, thereby enhancing the credibility of the findings. Beyond the C-index and calibration, decision curve analysis directly addressed the clinically fundamental question of whether using this model benefits patients, elevating model evaluation from statistical performance alone to the level of clinical decision support.

Several limitations should nevertheless be acknowledged. First, as a retrospective study, this analysis is inherently subject to risks of selection bias and information bias, and the completeness and accuracy of data collection may have influenced the validity and generalizability of the findings. Although independent validation was performed, the validation cohort was derived from the same geographic region (Shanghai) and a similar healthcare environment. The two cohorts likely shared comparable socioeconomic backgrounds, insurance coverage, and care pathways, resulting in relatively high population homogeneity. Accordingly, the reported performance metrics (e.g., C-index=0.78; AUCs 0.79–0.90) may still be somewhat optimistic and should be interpreted with appropriate caution. The generalizability of the model to broader populations, including those from different regions, healthcare settings, or racial and ethnic backgrounds, remains to be established in future multicenter prospective studies. In addition, the predictive scope of the model was limited to the first 14 postoperative days and therefore cannot inform the longer-term course of patients whose seroma had not resolved by day 14. Future work should extend the follow-up period and adopt prospective, multicenter, and geographically diverse designs to establish a more comprehensive prognostic model.

Second, this study incorporated only baseline and early postoperative variables and did not include potentially informative biological markers (such as gene expression profiles or inflammatory mediators) or psychosocial factors (such as psychological status and social support). These factors may play important roles in postoperative recovery among patients with breast cancer. Future studies should incorporate multidimensional variables to achieve a more comprehensive assessment of prognostic risk and to develop dynamic models with enhanced predictive performance.

## Conclusions

This study developed and validated a precise and clinically practical nomogram for individualized prediction of postoperative seroma resolution in patients with breast cancer. The model demonstrated clear clinical utility at multiple postoperative decision points and may facilitate the identification of patients at high risk for delayed seroma resolution, thereby enabling more efficient allocation of medical resources and more targeted implementation of interventions, with the ultimate goal of improving postoperative care. Future work should focus on prospective validation of the model and its integration into routine clinical workflows.
